# COVID-19 literature surveillance—A framework to manage the literature and support evidence-based decision-making on a rapidly evolving public health topic

**DOI:** 10.14745/ccdr.v49i01a02

**Published:** 2023-01-05

**Authors:** Tricia Corrin, Dima Ayache, Austyn Baumeister, Kaitlin Young, Kusala Pussegoda, Rukshanda Ahmad, Lisa Waddell

**Affiliations:** 1 Public Health Risk Sciences Division, National Microbiology Laboratory, Public Health Agency of Canada, Winnipeg, MB; 2 Centre for Food-borne, Environmental and Zoonotic Infectious Diseases, Infectious Disease Prevention Branch, Public Health Agency of Canada, Ottawa, ON

**Keywords:** COVID-19, evidence-based decision-making, literature surveillance, evidence synthesis, knowledge synthesis, SARS-CoV-2

## Abstract

**Background:**

The coronavirus disease 2019 (COVID-19) pandemic has led to a rapid surge of literature on severe acute respiratory syndrome coronavirus 2 and the wider impacts of the pandemic. Research on COVID-19 has been produced at an unprecedented rate, and the ability to stay on top of the most relevant evidence is top priority for clinicians, researchers, public health professionals and policymakers. This article presents a knowledge synthesis methodology developed and used by the Public Health Agency of Canada for managing and maintaining a literature surveillance system to identify, characterize, categorize and disseminate COVID-19 evidence daily.

**Methods:**

The Daily Scan of COVID-19 Literature project comprised a systematic process involving four main steps: literature search; screening for relevance; classification and summarization of studies; and disseminating a daily report.

**Results:**

As of the end of March 2022 there were approximately 300,000 COVID-19 and pandemic-related citations in the COVID-19 database, of which 50%–60% were primary research. Each day, a report of all new COVID-19 citations, literature highlights and a link to the updated database was generated and sent to a mailing list of over 200 recipients including federal, provincial and local public health agencies and academic institutions.

**Conclusion:**

This central repository of COVID-19 literature was maintained in real time to aid in accelerated evidence synthesis activities and support evidence-based decision-making during the pandemic response in Canada. This systematic process can be applied to future rapidly evolving public health topics that require the continuous evaluation and dissemination of evidence.

## Introduction

The rapid spread of severe acute respiratory syndrome coronavirus 2 (SARS-CoV-2), the virus that causes coronavirus disease 2019 (COVID-19), across the globe in early 2020 sparked an immediate urgency for evidence to understand the virus and how to combat it. Although evidence was scarce at the beginning of 2020, it was anticipated that a rapid surge of research would emerge based on recent experience with the Zika pandemic ((1,2)). As the pandemic began to unfold, there was a swift influx of evidence on COVID-19, which varied in breadth from clinical epidemiology to the impact of public health interventions. Staying on top of the most recent evidence was extremely important to keep researchers and decision-makers up-to-date as fast as possible on what was both known and unknown.

With the world looking for answers about the evolving COVID-19 pandemic, it was evident there was an urgent need for an efficient literature surveillance system to identify, manage, synthesize and disseminate this evidence daily. The aim of this article is to report the process developed by the Public Health Agency of Canada (PHAC) to manage and maintain a COVID-19 literature surveillance system on a predictable 24-hour cycle to meet the evidence needs of several research and policy groups while reducing redundancies in search and retrieval efforts.

## Methods

### Role of the Public Health Agency of Canada in response to the COVID-19 pandemic

The Public Health Agency of Canada is responsible for preparing and responding to public health emergencies ((3)). In response to the pandemic, an Emerging Sciences Group was created within PHAC consisting of employees at the Agency with a variety of expertise in infectious disease modelling, epidemiology, clinical care, diagnostics, virus research and knowledge synthesis expertise. The role of the Emerging Sciences Group was to help coordinate and share information across disciplines within the Agency and COVID-19 literature surveillance was a key priority identified by this group. In collaboration with Emerging Sciences Group members, a knowledge synthesis team with extensive experience in infectious diseases within PHAC took the lead in developing and maintaining a COVID-19 literature surveillance system to aid in the COVID-19 pandemic response.

### Purpose and scope of the project

The literature surveillance project was initiated in January 2020, with the main goal of creating and maintaining a central repository of COVID-19 literature to aid in evidence synthesis activities, promote the use of up-to-date evidence by researchers and policymakers through daily updates on new evidence, and support evidence-based decision-making across the Agency. This project was named the Daily Scan of COVID-19 Literature (referred to as the Daily Scan).

At its inception, there were no other repositories anywhere in the world that compiled the literature on COVID-19. Over time, several COVID-19 repositories were created (e.g. Cochrane, LOVE, LitCOVID, WHO COVID literature database), each with its own focus and methods for mapping the COVID-19 literature ((4–7)). This Daily Scan project differs in that 1) articles were searched and retrieved from preprint databases, which required manual extraction of citations, 2) all COVID-19 literature was included without restriction, 3) the search was conducted each morning from Monday to Friday (24-hour cycle) and 4) the literature was compiled and disseminated to end users daily.

**Figure 1:**
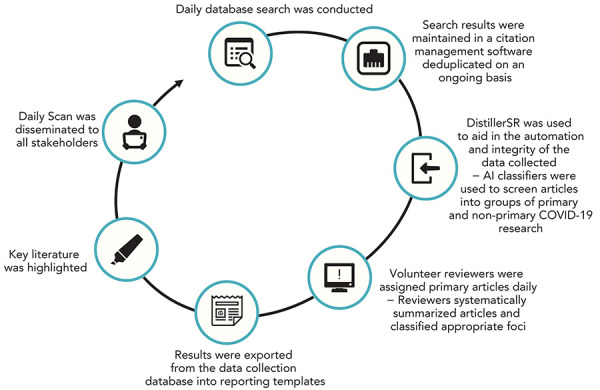
Flow chart of the Daily Scan process Abbreviations: AI, artificial intelligence; COVID-19, coronavirus disease 2019

The Daily Scan was developed based on well-known and established knowledge synthesis methodologies that employ systematic methods to identify, collect, map and report on evidence underpinning a broad topic, while also addressing the need for a real-time living evidence base that could be tapped into to monitor the evolution of priority topics ((8–12)). The Daily Scan comprised a systematic process involving four main steps: searching the literature, relevance screening; classification and summarization of studies; and disseminating evidence in a daily report (**Figure 1**). A high-level overview of each step in this process is described below and additional details can be found in **supplemental material**.

### Daily Scan team

Since the inception of this project, the National Microbiology Laboratory knowledge synthesis team has coordinated the contributions of more than 50 people from across PHAC. A search specialist and three scan leads managed and coordinated day-to-day project activities with oversight by a manager. Each day approximately 10–15 reviewers contributed to reviewing, categorizing and summarizing the literature on a part-time basis (1–3 hours a day).

### Search strategy

A search of the literature (published and pre-published) was conducted daily Monday through Friday. Searches to retrieve relevant COVID-19 literature were conducted in seven databases: PubMed, Scopus, BioRxiv, MedRxiv, ArXiv, SSRN and Research Square. ArXiv, SSRN and Research Square required a manual citation extraction process.

### Database management

Search results were managed in citation management software, Endnote (EndNote X8, Clarivate Analytics). To facilitate access to the citation database by end users, the citations were transferred to another citation management software, RefWorks (RefWorks, ProQuest LLC). The citations were then imported into the web-based systematic review management software DistillerSR^®^ (Evidence Partners Inc., Ottawa, Canada). Duplicates were identified and removed in each software.

Management of citations for screening, characterizing, and data extraction was conducted using DistillerSR. This platform allowed a large number of reviewers to work on the project simultaneously across the Agency.

### Categorization of COVID-19 literature

After the daily search results were imported into DistillerSR, two artificial intelligence classifiers (DistillerAI) were applied to automatically categorize the COVID-19 literature into three pre-defined groups: synthesis research (e.g. systematic reviews, meta-analyses, scoping reviews); primary research; and non-primary articles (e.g. narrative reviews, commentaries, letters to editors). The primary research then proceeded to a data collection level for further categorization and summarization.

Each morning, the primary literature was divided and distributed within DistillerSR to a team of reviewers. A data collection form designed at the beginning of the project was used to collect information for each primary research article in DistillerSR. The form allowed a reviewer to provide a brief synopsis of the evidence, classify it into predetermined foci (e.g. epidemiology, transmission) and ensure that there was a working article link to the article so it could be readily accessed. The reviewers also identified any primary literature that was noteworthy in their assigned articles to include in the “highlights section” of the Daily Scan email report to draw end-user attention to new research on priority COVID-19 topics. One reviewer filled out the data collection form for each article. The scan leads conducted frequent spot checks to verify results and ensure consistency.

## Results

### Volume and focus of literature

**Figure 2:**
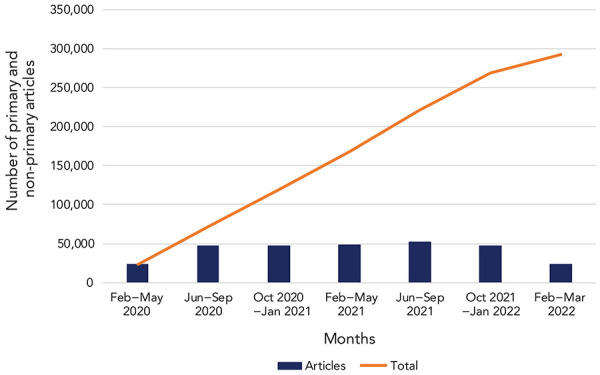
Volume of COVID-19 literature February 2020–March 2022

Initially there were fewer than 50 citations identified daily, which increased quickly through the winter of 2020 to a median of 700 citations per day. After the first six months of the pandemic (January–June 2020) the volume of research being produced stabilized at approximately 50,000 articles per six-month period (**Figure 2**). At of the end of March 2022 there were approximately 300,000 COVID-19 and pandemic-related citations in the database.

Although there was a median of 700 citations identified daily, the range was 200–2,500 citations. Generally, 50%–60% of the citations were primary research and the others were non-primary articles (e.g. literature reviews, science news, conference proceedings).

Reviewers tagged each primary research article with one or multiple pre-determined foci that were addressed by the outcomes in the article. At the end of March 2022, the total number of articles by foci included clinical data (n=48,179), epidemiology (n=42,622), healthcare response (n=17,559), public health response (n=15,505), coronavirology (n=14,480), mental health (n=11,261), modelling (n=11,184), therapeutics (n=10,946), diagnostics (n=10,532), immunology (n=10,357), public health interventions (n=8,737), vaccine research (n=8,373), transmission (n=4,547), long-term sequelae (n=2,376), animal model (n=2,358), infection prevention and control (n=1,935), randomized controlled trials (n=1,547), surveillance (n=1,291), economics (n=1,020), zoonoses/COVID-19 in animals (n=419) and other COVID-19/pandemic impact research not covered by other foci (n=15,811).

### Dissemination of the Daily Scan

The data was then compiled each day to produce the Daily Scan. The data was first exported out of DistillerSR into a Microsoft Excel template that was developed to automatically organize the data into the format required for the report. A Microsoft Word template was then populated with each article, foci(s) and summary. Finally, this report was placed in an email along with the literature highlights for the day and a link to both the citation database and Microsoft Excel spreadsheet accessible through Google Drive that contained a searchable list of all the literature with their foci categorizations and summaries. The mailing list for the Daily Scan included over 200 recipients and groups including federal, provincial and local public health agencies, and academic institutions.

### Database maintenance

As pre-published articles (preprints) underwent peer review and were published, they were picked up in the search and flagged. A process was developed to remove preprints from the databases once an article was published to ensure that preprint articles were not accidentally used in place of published articles by end users. The search specialist identified published articles that were previously preprints and proceeded to quarantine them in Refworks, DistillerSR and the Excel sheets.

### Utilization of the Daily Scan

The results of the Daily Scan were used in a variety of ways by end users across PHAC and other organizations. First, the daily report and highlights were used extensively by groups working on the pandemic response to keep apprised of key new research relevant to their work. As our database covered all COVID-19 literature, including wider consequences of the pandemic, it was a resource that met the needs of a wide range of end users responding to the pandemic.

Second, evidence synthesis teams across PHAC have capitalized on this database resource to rapidly respond to urgent requests for evidence, conduct rapid evidence syntheses on priority questions, and to streamline evidence into other domain-focused projects (e.g. vaccines, therapeutics, public health measures). The knowledge synthesis team that led this project has also conducted over 150 rapid evidence syntheses on high-priority COVID-19 topics since February 2020. An up-to date and searchable repository was a key feature of this project for producing these evidence synthesis products rapidly. Other groups also utilized the repository for evidence synthesis reviews, reporting evidence highlights to senior management, populating predictive models, creating guidelines, web content and answering media requests.

In November 2021, a short survey was sent to all the Daily Scan recipients to gain additional insight on how the Daily Scan was being utilized and to assess the need to continue this project. The survey reported a consensus that the Daily Scan remained a valuable and necessary tool for the ongoing pandemic response within and outside of PHAC. Several teams indicated that they would be negatively affected should it be discontinued and reported that they would have to allocate resources to search the literature to continue with their ongoing COVID-19 projects and activities.

## Discussion

The breadth of this project, the inclusion of all COVID-19 literature, and the 24-hour update cycle are unique to this project and key to its usefulness to the extensive needs of researchers and decision makers within and outside PHAC. Keeping dedicated expertise on the COVID-19 literature facilitated rapid response work and ongoing assessment of knowledge gaps during the pandemic.

## Limitations

There were several limitations to this methodology. First, this project was created and implemented within a very short timeframe using existing resources. Different software and further automation of searching, screening, classification of articles and reporting could have made this process more efficient. However, time and resource constraints with this project and other COVID-19 priority projects limited the capacity to keep the Daily Scan running while making significant changes that may have improved the efficiency of the process.

The process was developed to be feasible to conduct on a 24-hour cycle. This meant that while the search was thorough, we may have missed citations due to language or omission by the search strategy (Supplemental material). There was always the potential for human error in conducting the search, reviewing and classifying articles, and maintaining and updating the database.

### Current and future applications

Given the urgent need for evidence-based public health decisions during a public health emergency such as the COVID-19 pandemic, the rapid and systematic gathering and synthesis of evidence is extremely important. At the time of writing, this database continues to be maintained and remains an essential resource to multiple departments throughout PHAC for use in the continued COVID-19 response.

To be prepared for the next public health emergency, post-pandemic planning will be essential to improve upon our existing literature surveillance framework. A priority will be to increase automation and efficiency at different stages of the literature surveillance process by acquiring the software and expertise to embed more automation within the process. Development of a web interface or dashboard for the Daily Scan would reduce the amount of time spent creating and disseminating the reports. This would allow end users to directly access and interact with the COVID-19 literature database, eliminating many report preparation steps in the current process. As artificial intelligence technologies are rapidly evolving, future literature surveillance may be able to work towards automated study summarization, significantly reducing the human resources required to run the project. Any of the above possible improvements will increase efficiency of the process, making it more feasible to implement for the next public health emergency.

## Conclusion

This article provides insight into a process for developing and maintaining a literature surveillance system to manage the surge of COVID-19 research during the pandemic. Despite the unprecedented amount of literature on the pandemic, the literature surveillance process identified, characterized, summarized and disseminated evidence daily for over two years and facilitated the use of evidence in decision-making by PHAC and external stakeholders. This framework could be applied to any public health emergency with a rapidly evolving evidence base that requires ongoing real-time monitoring for use in decision-making.

## Supplemental material

These documents can be accessed on the Supplemental material file.
